# Multifunctional Hydrogels for Advanced Cancer Treatment: Diagnostic Imaging and Therapeutic Modalities

**DOI:** 10.3390/gels11060426

**Published:** 2025-06-01

**Authors:** Kyung Kwan Lee, Kwangmo Go, Eonjin Lee, Hongki Kim, Seonwook Kim, Ji-Hyun Kim, Min Suk Chae, Jin-Oh Jeong

**Affiliations:** 1 Wake Forest Institute for Regenerative Medicine, Wake Forest School of Medicine, Winston-Salem, NC 27157, USA; kylee@wakehealth.edu; 2 Department of Chemistry, Kongju National University, Kongju 32588, Republic of Korea; hongkikim@kongju.ac.kr; 3 Department of Chemistry, Carnegie Mellon University, 4400 Fifth Avenue, Pittsburgh, PA 15213, USA; kwangmog@andrew.cmu.edu; 4 Bionanotechnology Research Center, Korea Research Institute of Bioscience and Biotechnology (KRIBB), Daejeon 34141, Republic of Korea; eonjin@kribb.re.kr; 5 Earth Environment Research Center, Kongju National University, Gongju-si 32588, Republic of Korea; 6 Department of Internal Medicine, Wake Forest School of Medicine, Winston-Salem, NC 27157, USA; seokim@wakehealth.edu; 7 Division of Gastroenterology, Department of Internal Medicine, College of Medicine, The Catholic University of Korea, Seoul 06591, Republic of Korea; dktkfpf82@gmail.com; 8 Department of Anesthesiology and Pain Medicine, Seoul St. Mary’s Hospital, College of Medicine, The Catholic University of Korea, Seoul 06591, Republic of Korea

**Keywords:** hydrogel, cancer, theranostics, treatment, diagnosis

## Abstract

Multifunctional hydrogels represent an emerging technological advancement in cancer therapeutics, integrating diagnostic imaging capabilities with therapeutic modalities into comprehensive, multifunctional systems. These hydrogels exhibit exceptional biocompatibility, biodegradability, high water retention capacity, and tunable mechanical properties, enabling precise drug delivery while minimizing systemic side effects. Recent innovations in stimuli-responsive components facilitate intelligent, controlled drug release mechanisms triggered by various stimuli, including changes in pH, temperature, magnetic fields, and near-infrared irradiation. Incorporating diagnostic imaging agents, such as magnetic nanoparticles, fluorescent dyes, and radiolabeled isotopes, substantially improves tumor visualization and real-time therapeutic monitoring. Multifunctional hydrogels effectively integrate chemotherapy, photothermal therapy, photodynamic therapy, immunotherapy, and their synergistic combinations, demonstrating superior therapeutic outcomes compared to conventional methods. Particularly, injectable and in situ-forming hydrogels provide sustained local drug delivery postoperatively, effectively reducing tumor recurrence. However, challenges persist, including initial burst release, mechanical instability, regulatory barriers, and scalability concerns. Current research emphasizes advanced nanocomposite formulations, biofunctionalization strategies, and innovative manufacturing technologies like 3D bioprinting to facilitate clinical translation. This review comprehensively summarizes recent advancements, clinical applications, and future perspectives of multifunctional hydrogel systems for enhanced cancer treatment, underscoring their potential to revolutionize personalized oncology.

## 1. Introduction

Cancer remains a critical global health challenge and one of the leading causes of death worldwide, posing considerable therapeutic difficulties despite significant advancements in clinical oncology. In 2020, approximately 19.3 million new cancer cases and nearly 10 million cancer-related deaths were reported globally, underscoring an urgent demand for more precise, effective, and advanced therapeutic strategies [1,2,3]. Conventional cancer treatment modalities, including surgical resection, chemotherapy, radiation therapy, and immunotherapy, have substantially improved patient survival and quality of life. Nevertheless, these traditional approaches exhibit significant limitations, such as systemic toxicity, insufficient specificity, multidrug resistance, recurrence, and severe side effects on healthy tissues [4,5,6,7,8]. Chemotherapy and radiation treatments, for instance, often damage rapidly dividing normal cells, causing pronounced side effects, impaired immune function, and diminished patient well-being. Additionally, surgical interventions, despite their efficacy in tumor removal, frequently risk incomplete resection, leaving behind residual microscopic cancer cells that can trigger recurrence or metastasis [9,10,11,12,13].

In response to these clinical challenges, significant research efforts have been directed toward developing multifunctional hydrogel-based systems capable of delivering targeted and sustained therapeutic effects integrated with real-time diagnostic imaging—a concept commonly referred to as “theranostics” [14,15,16,17]. Multifunctional hydrogels are three-dimensional polymer networks engineered to encapsulate and release therapeutic agents in a controlled and localized manner directly at tumor sites. This targeted approach significantly minimizes systemic exposure and adverse effects [18,19,20,21]. Due to their advantageous physicochemical properties, including excellent biocompatibility, biodegradability, high water content, adjustable mechanical properties, and structural similarity to natural extracellular matrices, multifunctional hydrogels are ideal biomaterials for various cancer therapies. These hydrogels are not only effective carriers of conventional chemotherapeutic agents but also serve as versatile platforms for advanced treatments such as photothermal therapy (PTT), photodynamic therapy (PDT), immunotherapy, and gene therapy [22,23,24,25,26].

Furthermore, multifunctional hydrogels offer the unique advantage of integrating diagnostic imaging modalities, enabling clinicians to monitor drug distribution, therapeutic response, and tumor progression in real-time. Hydrogel-based systems incorporating imaging agents such as magnetic nanoparticles for magnetic resonance imaging (MRI), fluorescent dyes for fluorescence (FL) imaging, gold nanoparticles for computed tomography (CT), and echogenic microbubbles for ultrasound (US) imaging have been extensively explored [27,28,29,30,31]. Additionally, emerging imaging techniques—including photoacoustic (PA) imaging, Raman spectroscopy, and narrow-band spectroscopy—have been successfully integrated into hydrogel platforms. These novel approaches significantly enhance imaging resolution, tissue penetration, and diagnostic accuracy, thereby improving tumor localization and the monitoring of therapeutic efficacy and guiding clinical decisions [32,33,34,35].

One of the most notable advantages of multifunctional hydrogels is their stimuli-responsive nature. Such hydrogels can dynamically alter their structure and functional characteristics in response to internal stimuli (such as pH variations, enzymes, hypoxia, and redox potentials) or external triggers (temperature fluctuations, magnetic fields, US, and near-infrared (NIR) irradiation), precisely controlling drug release and therapeutic activation [36,37,38,39,40,41,42,43,44,45]. For instance, thermoresponsive hydrogels based on poly(N-isopropylacrylamide) (PNIPAAm) transform from a liquid (sol) to a solid (gel) state at physiological temperatures, enabling minimally invasive delivery directly into tumors with sustained local drug release [46]. Similarly, pH-responsive hydrogels exploit the acidic tumor microenvironment to selectively release anticancer agents, enhancing therapeutic efficacy and reducing harm to healthy tissues [47]. Magnetic-responsive hydrogels containing iron oxide nanoparticles facilitate remote, noninvasive control over drug release while providing simultaneous imaging capabilities, dramatically improving therapeutic precision and patient compliance [48].

In addition to conventional design principles, multifunctional hydrogels have also been increasingly developed to respond to specific biological cues in the tumor microenvironment, such as hypoxia, acidic pH, and elevated enzymatic activity. Hypoxia-responsive hydrogels incorporate functional groups that undergo cleavage or physicochemical transformation under low-oxygen conditions—a hallmark of many solid tumors. These systems exploit the overexpression of hypoxia-inducible factors and limited oxygen diffusion in tumors to achieve spatially confined therapeutic activation. pH-responsive hydrogels are engineered to respond to the mildly acidic extracellular environment of tumors (typically pH 6.5–6.8) relative to normal tissues (pH 7.4) through the use of acid-labile linkers or protonatable moieties that trigger swelling, degradation, or drug release. Enzyme-responsive hydrogels, on the other hand, are formulated to degrade in the presence of tumor-associated proteases such as matrix metalloproteinases. These systems often incorporate cleavable peptide-based crosslinkers or substrates, enabling site-specific hydrogel breakdown and on-demand drug delivery. Collectively, these tumor microenvironment-responsive mechanisms offer enhanced selectivity and therapeutic precision, positioning multifunctional hydrogels as attractive candidates for personalized cancer treatment.

Injectable hydrogels have gained considerable attention for their minimally invasive administration, significantly improving patient comfort and enabling repeat administrations without surgery. In situ gelation mechanisms further simplify clinical use, providing sustained local therapeutic release postoperatively, thus reducing the risk of tumor recurrence, promoting wound healing, and facilitating tissue regeneration [49,50]. Despite these promising developments, challenges such as initial burst release of drugs, maintaining hydrogel stability and mechanical integrity under physiological conditions, regulatory approval complexities, and scalability in manufacturing remain significant obstacles. Nevertheless, ongoing advancements in bioengineering, material science, and nanotechnology, including biofunctionalization, optimized crosslinking chemistries, advanced nanomaterials, 3D bioprinting, and artificial intelligence (AI)-driven personalized medicine, continue to offer promising solutions [51,52,53].

**Figure 1:**
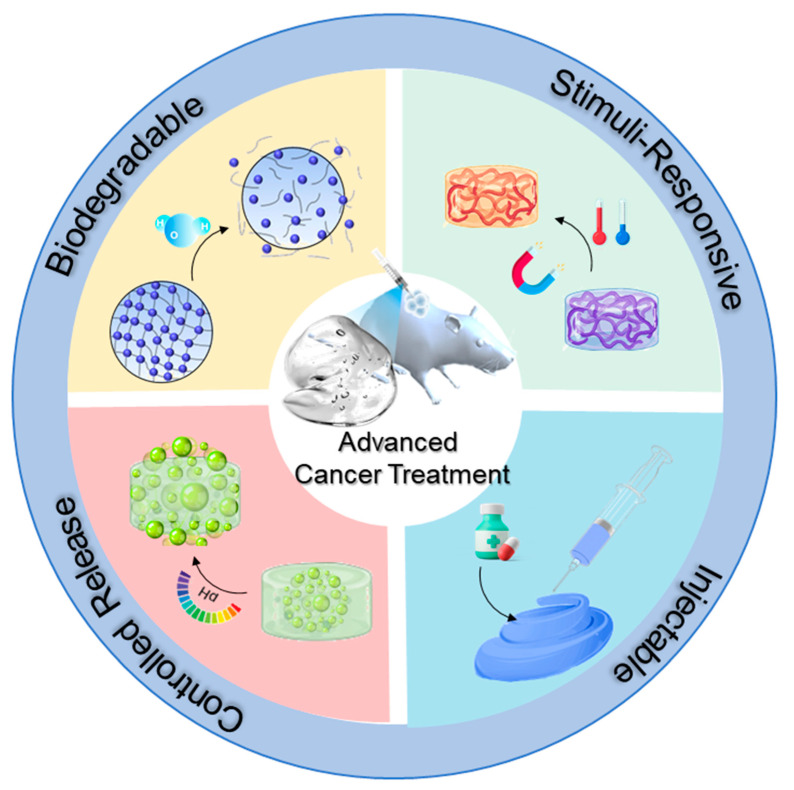
Strategy and mechanism of a multifunctional hydrogel for advanced cancer therapy.

This comprehensive review systematically discusses the latest advances in multifunctional hydrogels for cancer theranostics, beginning with their fundamental characteristics, classifications, and physicochemical properties (Figure 1). Subsequently, it explores recent developments in integrating diagnostic imaging techniques for improved cancer detection and treatment monitoring. It also evaluates various therapeutic modalities facilitated by multifunctional hydrogels, including chemotherapy, PTT, PDT, immunotherapy, and combination therapies. Finally, this review examines clinical applications, identifies current challenges and limitations, and outlines future directions and opportunities, aiming to inspire continued interdisciplinary research toward safer, more effective, and personalized cancer management.

## 2. Fundamentals and Characteristics of Multifunctional Hydrogels

### 2.1. Definition and Classification

Hydrogels are three-dimensional polymeric networks capable of absorbing and retaining substantial amounts of water, closely mimicking the physical and structural characteristics of biological tissues. Their exceptional biocompatibility, biodegradability, and controlled drug-delivery capabilities position them as ideal biomaterials for various biomedical applications, particularly in cancer treatment [54,55]. Multifunctional hydrogels are broadly classified based on their polymeric origins: natural, synthetic, and hybrid hydrogels (Table 1). Natural hydrogels are derived from biopolymers such as alginate, hyaluronic acid, collagen, and chitosan. These materials exhibit excellent biocompatibility, minimal immunogenicity, and bioactivity beneficial for tissue regeneration and wound healing [56,57,58,59,60,61,62]. Synthetic hydrogels, including polyethylene glycol (PEG), poly(lactic-co-glycolic acid) (PLGA), polyvinyl alcohol (PVA), and poly(N-isopropylacrylamide) (PNIPAAm), offer tailored mechanical properties, controlled degradation rates, and specific stimuli-responsive behaviors, making them highly adaptable for targeted clinical applications [63,64,65,66,67,68,69,70,71]. Hybrid hydrogels combine the advantages of both natural and synthetic polymers, enabling precise optimization of mechanical strength, biological performance, and responsiveness, which are particularly valuable in precision oncology and patient-specific therapeutic strategies [72,73,74].

### 2.2. Physicochemical Properties

The physicochemical properties of multifunctional hydrogels critically influence their performance in cancer treatment applications. Essential properties include biocompatibility, controlled biodegradability, stimuli responsiveness, mechanical integrity, and regulated drug-release profiles (Table 2) [75,76,77]. The highly porous, hydrophilic nature of these hydrogels allows for efficient encapsulation and prolonged release of therapeutic agents. Stimuli-responsive hydrogels exhibit dynamic reactions to specific biological or external stimuli, such as pH variations, temperature fluctuations, magnetic fields, or NIR irradiation. This adaptability facilitates precise targeting and controlled drug delivery directly to tumor tissues, thereby enhancing therapeutic effectiveness while minimizing systemic toxicity [78,79,80,81,82,83,84,85,86,87,88,89,90,91,92]. Additionally, hydrogel mechanical properties can be fine-tuned to emulate the physical characteristics of tumor microenvironments, further optimizing their integration and therapeutic efficacy in cancer management.

## 3. Preparation of Multifunctional Hydrogels

The preparation methods of hydrogels significantly influence their structural, mechanical, and functional characteristics, all of which are crucial for biomedical applications, particularly in cancer therapy. These methods are broadly classified into physical crosslinking, chemical crosslinking, and emerging advanced techniques such as photo-crosslinking and 3D bioprinting.

Physically crosslinked hydrogels are formed through non-covalent interactions, including hydrogen bonding, ionic interactions, crystallization, and hydrophobic interactions. These methods are attractive for biomedical use because they typically avoid the use of toxic crosslinking agents and operate under mild conditions. For instance, ionic crosslinking with divalent cations like calcium can induce gelation in alginate-based systems, making them suitable for drug encapsulation and local delivery. Similarly, freeze–thaw cycles are commonly used to induce gelation in PVA-based hydrogels by promoting crystalline regions [93].

Chemical crosslinking involves covalent bond formation between polymer chains, resulting in stable, robust hydrogel networks. This approach allows precise control over the hydrogel’s network structure, mechanical strength, and degradation rate. Common strategies include radical polymerization (initiated thermally or photochemically), Schiff base reactions, thiol-ene reactions, and enzymatic crosslinking. While chemically crosslinked hydrogels typically exhibit enhanced stability and tunability, they may involve reactive species or conditions that require careful optimization to maintain biocompatibility, especially when used for in vivo applications [94,95,96].

Photo-crosslinking has emerged as a powerful tool to spatially and temporally control hydrogel formation. This technique uses light (usually UV or visible) in the presence of photo-initiators to trigger rapid polymerization. It is particularly valuable in biomedical settings requiring in situ gelation, such as injectable systems or light-guided therapeutic delivery. Furthermore, it supports complex patterning in tissue engineering [97].

Recent developments have introduced 3D bioprinting as an advanced hydrogel fabrication method. This technique allows for the precise layer-by-layer deposition of cell-laden hydrogel bio-inks to build complex, patient-specific scaffolds with controlled architecture, porosity, and drug distribution. The integration of 3D bioprinting and hydrogel technology is accelerating the development of personalized cancer therapies and tumor models for drug screening [98,99].

Overall, the selected preparation method directly impacts a hydrogel’s physicochemical properties, including its porosity, mechanical integrity, degradation kinetics, and drug release profile. Therefore, the fabrication technique must be carefully matched with the intended biomedical application to ensure optimal therapeutic performance.

## 4. Design Strategies for Diagnostic Imaging Hydrogels

**Figure 2:**
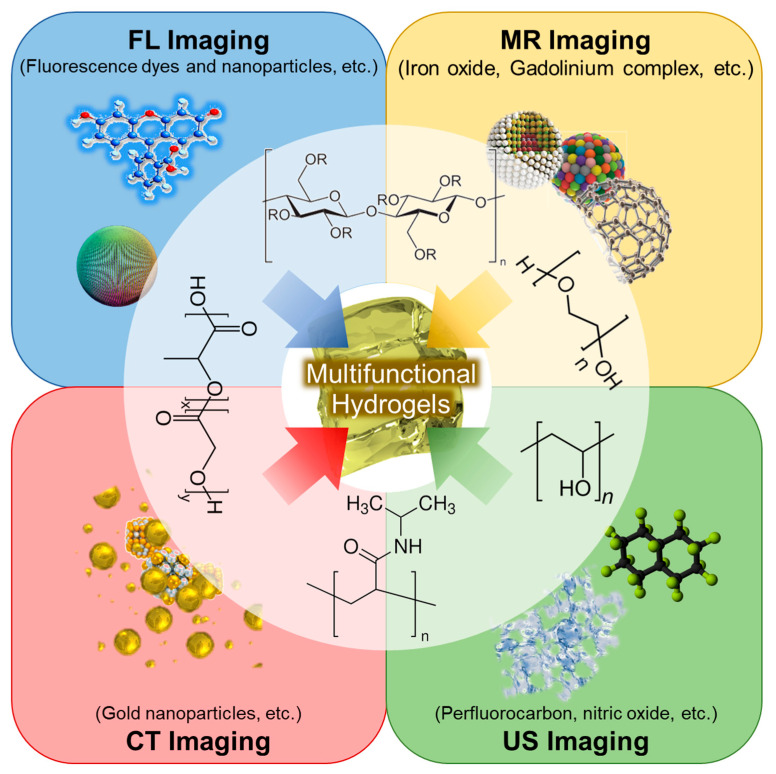
Schematic representation of multifunctional hydrogel-based strategies integrating diverse polymer platforms and imaging modalities for cancer diagnosis.

The integration of multifunctional hydrogels with advanced diagnostic imaging techniques significantly enhances cancer detection, diagnosis, and therapeutic monitoring (Figure 2). Each imaging modality leverages specific hydrogel properties to improve imaging sensitivity, resolution, and real-time visualization (Table 3).

### 4.1. MRI

MRI is a widely used non-invasive diagnostic modality that offers high spatial resolution and excellent soft tissue contrast, making it particularly advantageous for detecting and monitoring tumor progression. The integration of MRI capabilities into hydrogel systems has enabled the development of theranostic platforms that allow for real-time imaging in conjunction with localized therapeutic delivery [100].

*T*_1_-weighted MRI contrast enhancement is commonly achieved through the incorporation of gadolinium(III) (Gd^3+^)-based compounds, which produce bright signal intensities and facilitate high-resolution visualization of hydrogel depots in vivo. Hydrogel matrices functionalized with chelating ligands, such as DTPA or Gd-DOTA, have demonstrated stable Gd^3+^ retention and sustained signal generation following subcutaneous or intratumoral administration [101]. These hydrogels often exhibit favorable biodegradability, biocompatibility, and physical stability, enabling their long-term use for therapeutic monitoring without eliciting adverse tissue reactions [102].

In addition to conventional *T*_1_-weighted imaging, Chemical Exchange Saturation Transfer (CEST) MRI has emerged as a promising label-free imaging technique. CEST MRI enhances image contrast based on the exchange of protons between water and endogenous or exogenous metabolites. Hydrogels containing exchangeable functional groups, such as hydroxyl or amine moieties, can generate CEST contrast without requiring external contrast agents [103,104]. These CEST-active hydrogels enable sensitive tracking of hydrogel degradation, spatial localization, and drug release behavior under physiological conditions [105]. Furthermore, MRI-compatible hydrogels have been engineered to avoid imaging artifacts in *T*_2_-weighted sequences, particularly in soft tissue environments such as the brain. Hydrogels with optimized composition and mechanical properties conform to complex anatomical surfaces while maintaining imaging fidelity. These systems not only support diagnostic imaging but also offer functionalities such as electrical conductivity or therapeutic agent loading, extending their utility beyond passive imaging substrates [106,107].

Altogether, MRI-responsive hydrogels offer a powerful tool for image-guided cancer management, combining diagnostic imaging, spatial tracking, and therapeutic delivery within a single platform. Their ability to provide dynamic feedback in real time enhances precision in cancer treatment and supports the development of responsive, minimally invasive theranostic systems.

### 4.2. FL and Optical Imaging

FL and optical imaging modalities offer high sensitivity and real-time visualization for intraoperative guidance and cancer diagnosis. Hydrogel platforms have been increasingly engineered to incorporate fluorescent probes, enabling site-specific visualization of tumors and real-time tracking of hydrogel degradation. Injectable hydrogels embedded with quantum dots or small molecule dyes enable stable, long-term fluorescence imaging in vivo. These systems are especially effective for monitoring drug release, surgical margins, and tissue regeneration. Dual-channel fluorescent hydrogels have demonstrated the ability to simultaneously track material degradation and host tissue response, offering valuable insight for therapeutic applications [108,109,110]. To enhance spatial resolution and specificity, some hydrogel formulations utilize carbon dots or supramolecular nanofibers, such as fluorophores, which improve optical stability and biocompatibility. Furthermore, the integration of NIR dyes such as indocyanine green has enabled deeper tissue imaging with reduced background interference, making these hydrogels especially useful for FL-guided surgery [111,112,113,114,115,116].

Overall, fluorescence-integrated hydrogels represent a versatile and highly sensitive modality for cancer imaging, offering customizable visualization profiles and compatibility with multimodal theranostic approaches.

### 4.3. CT Imaging

CT provides high spatial resolution and rapid acquisition times, allowing detailed anatomical visualization. The incorporation of radiopaque materials into hydrogels has enabled their effective use as CT-visible imaging platforms.

Iodinated contrast agents or metallic nanoparticles such as gold and ytterbium have been successfully integrated into hydrogel matrices to enhance X-ray attenuation and visibility. These contrast-enhanced hydrogels allow precise localization of tumor boundaries and can be used preoperatively for image-guided interventions [117,118,119]. The use of spectral photon-counting CT and dual-energy techniques has further enabled the differentiation of hydrogel-embedded agents from surrounding tissues, facilitating more accurate diagnosis and treatment planning. These CT-responsive hydrogels are particularly advantageous for applications requiring both structural fidelity and real-time imaging.

### 4.4. US and PA Imaging

US imaging is a widely accessible, non-ionizing modality ideal for real-time image guidance. Hydrogel systems incorporating microbubbles or nanobubbles enhance echogenic contrast, enabling more precise localization and monitoring of drug delivery in tumor environments. Recent developments in ultrasound-responsive hydrogels have utilized bursting nanobubble agents that respond to acoustic pressure, enabling both diagnostic imaging and ultrasound-triggered therapeutic release. This dual functionality supports real-time feedback and controlled treatment delivery [120,121].

PA imaging, which combines optical and ultrasound modalities, benefits from hydrogels embedded with photo-absorbers such as carbon nanotubes or organic dyes. These hydrogels provide strong optical absorption and generate acoustic signals upon laser stimulation, enabling high-contrast imaging at greater tissue depths. This hybrid modality is particularly promising for neural stimulation and deep-tissue tumor imaging [122,123,124,125,126].

Together, ultrasound and PA-responsive hydrogels expand the functional landscape of cancer diagnostics, offering minimally invasive, image-guided, and stimuli-responsive therapeutic systems.

### 4.5. Others Imaging Techniques

Beyond traditional imaging methods, multifunctional hydrogels have been integrated with advanced imaging modalities such as Raman spectroscopy and narrow-band imaging. Raman spectroscopy-based hydrogels incorporate Raman-active nanoparticles (e.g., gold nanorods), enabling highly sensitive, label-free molecular imaging [127,128,129].

**Table 3:** Imaging modalities using multifunctional hydrogels for cancer treatment.

Imaging Modality	Hydrogel Types	Key Features	Materials	References
MRI (*T*_1_-weighted, CEST) imaging	Gd^3+^-DTPA/DOTA hydrogels, CEST-active hydrogel	High spatial resolution, real-time imaging, label-free options, artifact minimization	Gadolinium compounds, hydroxyl/amine-functional hydrogels	[100,101,102,103,104,105,106,107]
FL and Optical imaging	Quantum dot or dye-loaded injectable hydrogels; NIR dye-integrated systems	High sensitivity, real-time visualization, FL-guided surgery compatibility	Quantum dots, carbon dots, indocyanine green	[108,109,110,111,112,113,114,115,116]
CT imaging	Radiopaque hydrogels with metallic nanoparticles or iodine-based agents	High resolution, accurate tumor localization, spectral CT compatibility	Gold, ytterbium, iodinated compounds	[117,118,119]
US and PA imaging	Bubble-encapsulated hydrogels, photo-absorber embedded systems	Non-ionizing, dual diagnostic and therapeutic capabilities, deep-tissue imaging	Microbubbles, carbon nanotubes, organic dyes	[120,121,122,123,124,125,126]
Raman imaging	Raman-active hydrogel systems with embedded nanoparticles	Molecular specificity, label-free detection	Gold nanorods	[127,128,129]

## 5. Therapeutic Modalities Using Hydrogels

Multifunctional hydrogels function as sophisticated platforms for controlled, sustained therapeutic delivery, substantially enhancing cancer treatment outcomes. These hydrogels effectively support diverse therapeutic modalities, including chemotherapy, PTT, PDT, immunotherapy, and combination therapies (Table 4).

### 5.1. Chemotherapy

Chemotherapy-loaded hydrogels have emerged as a promising strategy to enhance the efficacy and specificity of conventional chemotherapeutics while minimizing systemic toxicity. These hydrogels provide sustained, localized release of anticancer agents such as doxorubicin (DOX), paclitaxel, and docetaxel (DTX), thereby maintaining therapeutic concentrations at tumor sites and reducing off-target effects. Thermosensitive and pH-responsive hydrogel systems, particularly those based on polypeptides, PLGA, and chitosan derivatives, have been developed to facilitate injectable in situ gelation and controlled drug release.

Multidrug delivery platforms combining hydrophilic and hydrophobic agents within a single hydrogel matrix have also been shown to significantly improve chemotherapeutic synergy and tumor suppression. For example, hydrogels incorporating DOX with micellar-encapsulated DTX have demonstrated enhanced antitumor efficacy and prolonged local retention, making them suitable for post-surgical cancer therapy [130,131,132,133,134,135].

### 5.2. PTT

Hydrogels designed for PTT integrate photo-absorbing agents, such as gold nanorods, polydopamine, or carbon-based nanomaterials, that convert NIR irradiation into localized heat. This thermal effect induces tumor ablation while sparing surrounding healthy tissues. Thermo-sensitive hydrogels serve as both delivery matrices and activatable therapeutic agents, enabling minimally invasive intra-tumoral injection and NIR-triggered activation.

These systems often achieve dual functionality, facilitating both PTT and controlled drug release. The spatial and temporal precision of hydrogel-mediated PTT contributes to improved safety profiles and treatment outcomes in localized solid tumors [136,137,138,139,140].

### 5.3. PDT

PDT utilizes light-activated photosensitizers to generate reactive oxygen species (ROS) that induce oxidative damage and apoptosis in tumor cells. Injectable hydrogels incorporating photosensitizers such as chlorin e6 (Ce6) or porphyrins have been designed to enhance ROS generation and treatment selectivity upon NIR light exposure.

These hydrogel systems offer the advantage of localized delivery and prolonged retention of photosensitizers at tumor sites, thereby improving light penetration efficiency and minimizing systemic side effects. Moreover, some hydrogels co-deliver photosensitizers with chemotherapeutics, enabling synergistic photo-chemo therapeutic approaches for enhanced tumor inhibition [141,142,143,144,145].

### 5.4. Immunotherapy

Multifunctional hydrogels have shown great potential in enhancing cancer immunotherapy by enabling localized, sustained delivery of immune checkpoint inhibitors (ICIs), cytokines (e.g., IL-2, IFN-γ), and tumor vaccines. These systems improve immune activation at tumor sites while reducing systemic toxicity and immune-related adverse events.

Hydrogels co-loaded with ICIs and chemotherapeutics or immune adjuvants have demonstrated enhanced antitumor immune responses through both direct cytotoxicity and immune modulation. Smart hydrogels that are responsive to tumor microenvironment stimuli (e.g., ATP levels or pH) further allow the synchronized release of immunotherapeutic agents to occur in response to biological cues, thereby maximizing immune engagement [146,147,148,149,150,151,152,153,154].

Collectively, hydrogel-based immunotherapeutic platforms represent a versatile approach to enhance cancer immunotherapy through localized, sustained, and stimuli-responsive delivery of immuneactive agents.

### 5.5. Combination Therapy

Combination hydrogels integrate multiple therapeutic strategies, such as chemo-photothermal or immunotherapy-phototherapy combinations, achieving synergistic effects beyond single-modality treatments [136,137,142,143,144,150]. This approach comprehensively addresses cancer heterogeneity, significantly improving therapeutic outcomes.

## 6. Applications of Multifunctional Hydrogels in Cancer Theranostics

Multifunctional hydrogels have significantly enhanced clinical cancer management by facilitating precise and minimally invasive therapeutic interventions, addressing challenges such as tumor recurrence, metastasis, and targeted drug delivery (Table 5) [155,156].

### 6.1. In Situ Injectable Hydrogels for Localized Cancer Therapy

**Figure 3:**
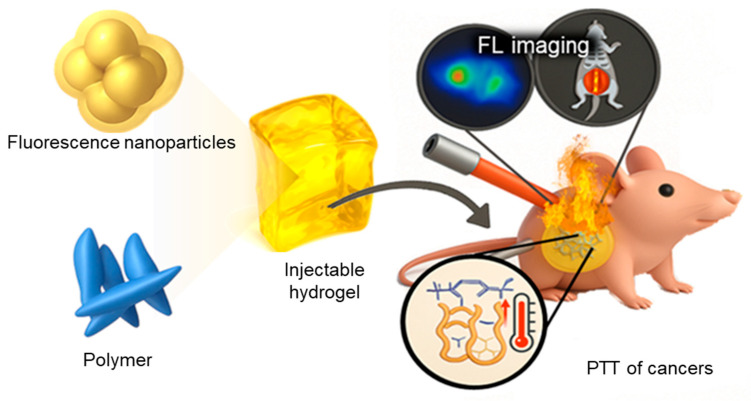
Schematic illustration of an injectable theranostic hydrogel composed of PVA integrated with fluorescent polydopamine for cancer FL imaging and PTT [162].

Injectable hydrogels capable of in situ gelation allow minimally invasive application directly at tumor sites, providing sustained localized drug delivery (Figure 3) [157,158,159,160,161]. These systems enhance therapeutic specificity, minimize systemic exposure, and improve patient compliance and treatment outcomes.

### 6.2. Hydrogels for the Prevention of Tumor Recurrence and Metastasis

Multifunctional hydrogels have effectively reduced postoperative tumor recurrence and metastasis by locally delivering chemotherapeutics or immunotherapeutic agents, maintaining high local drug concentrations, and effectively inhibiting residual tumor cell proliferation [163,164,165,166,167].

### 6.3. Hydrogels for Minimally Invasive Localized Treatment

Minimally invasive hydrogel systems, integrating both imaging and therapeutic agents, significantly improve treatment precision and patient comfort. This approach enables accurate tumor localization, real-time therapeutic monitoring, and controlled therapeutic release, which are particularly beneficial for sensitive or difficult-to-reach anatomical locations [168,169].

**Table 5:** Application of multifunctional hydrogel in cancer theranostics.

Application Area	Cancers	Key Features	Advantages	References
In Situ Injectable Hydrogels for Localized Cancer Therapy	Breast, Liver, Prostate	Thermo-responsive, pH-responsive hydrogels (e.g., chitosan, hyaluronic acid-based)	Minimally invasive, reduced systemic toxicity, targeted therapy	[158,159,160,161,162]
Prevention of Tumor Recurrence and Metastasis	Colorectal, Breast, Lung	Biodegradable hydrogels (e.g., PLGA, alginate-based)	Effective prevention of recurrence and metastasis, reduced side effects	[163,164,165,166,167]
Minimally Invasive Localized Treatment	Skin, Pancreatic, Head and Neck	Photo-responsive, Magnetic-responsive hydrogels (e.g., gold nanoparticles, iron oxide nanoparticles embedded)	Enhanced therapeutic accuracy, reduced invasiveness, real-time monitoring	[168,169]

## 7. Challenges and Future Perspectives

### 7.1. Current Limitations in Multifunctional Hydrogel Systems

Multifunctional hydrogels have demonstrated great potential in preclinical models for integrated cancer therapy and diagnosis. However, despite rapid advancements in their design and laboratory-scale performance, clinical translation remains limited. One of the primary obstacles is the lack of comprehensive clinical trial data validating their safety, efficacy, and reproducibility in human populations. Most systems remain confined to early-stage development, with only a few progressing beyond small animal models.

In particular, challenges related to regulatory approval, long-term biocompatibility, and scalable manufacturing significantly hinder the path toward clinical application. A few injectable hydrogel systems have successfully crossed the translational gap—most notably, Feraheme^®^ (ferumoxytol), a polysaccharide-based iron oxide nanoparticle formulation approved by the FDA for MRI contrast enhancement, and Tisseel^®^, a fibrin-based hydrogel widely employed for surgical tissue sealing. These examples demonstrate the clinical promise of hydrogel platforms but also underscore the stringent regulatory and safety standards that must be met for approval.

Beyond the need for clinical data, several intrinsic limitations of multifunctional hydrogels complicate their clinical readiness. Among the most pressing is the burst release phenomenon, where therapeutic agents are rapidly discharged immediately after administration. This uncontrolled release can reduce the intended sustained delivery profile, compromise therapeutic efficacy, and elevate systemic toxicity—particularly in chemotherapy-based applications.

Another concern is the in vivo mechanical and chemical stability of hydrogels. Many current formulations are prone to premature degradation or exhibit inconsistent behavior under physiological conditions, compromising their long-term therapeutic reliability. Effective cancer therapy demands hydrogels that can withstand mechanical stresses, maintain predictable degradation profiles, and ensure sustained drug release.

Navigating the regulatory landscape presents additional hurdles. Agencies such as the FDA and EMA require a clear classification of the hydrogel product, whether as a drug, device, or combination product, which significantly affects the scope of required clinical evidence and regulatory strategy. Multifunctional hydrogels must also undergo rigorous in vivo evaluations addressing biocompatibility, immunogenicity, degradation kinetics, and pharmacokinetics, all of which are critical for regulatory approval.

Furthermore, meeting Good Manufacturing Practice (GMP) standards is particularly challenging due to the complex architecture of these systems. Incorporating nanomaterials or stimuli-responsive components demands sterile, reproducible, and large-scale fabrication processes. Ensuring batch-to-batch consistency, quality control, and long-term storage stability remains a significant technical barrier. The inclusion of nanostructures often intensifies regulatory scrutiny, necessitating additional toxicological assessments and risk mitigation strategies [170,171,172].

In conclusion, while multifunctional hydrogels offer exciting opportunities for personalized and integrated cancer care, their clinical adoption depends on overcoming substantial barriers in regulatory classification, manufacturing scalability, biocompatibility validation, and clinical efficacy. Addressing these issues through standardized protocols and collaborative regulatory frameworks will be crucial for advancing multifunctional hydrogels from the bench to the bedside.

### 7.2. Future Directions

#### 7.2.1. Material Science Innovations and Advanced Hydrogel Designs

To overcome current barriers in the clinical translation of multifunctional hydrogels, continuous innovation in materials science and hydrogel design is essential. Future re-search should focus on finely tuning degradation kinetics, mechanical strength, and swelling behavior to ensure predictable and sustained drug release under physiological conditions. Recent advances in crosslinking strategies, including dynamic covalent bonding and supramolecular interactions, offer promising solutions for enhancing mechanical robustness, imparting self-healing properties and improving responsiveness to both internal and external stimuli. These features collectively support prolonged therapeutic efficacy and structural stability in vivo [173,174,175,176,177,178].

A particularly promising area involves the incorporation of stimuli-responsive cross-linkers, such as those sensitive to enzymes or pH. These elements are engineered to de-grade progressively in the tumor microenvironment, facilitating localized, on-demand drug release. This approach minimizes premature drug leakage while enabling site-specific therapeutic activation aligned with pathological cues.

In addition to chemical control, spatial compartmentalization strategies are gaining attention. Innovations such as layered or core–shell hydrogel architectures which consist of multiple hydrogel layers, each with distinct functionalities or release profiles, allow the spatially and temporally regulated delivery of therapeutic agents to occur. These designs limit early stage burst release and enhance the long-term control of therapeutic availability, improving efficacy and safety compared to homogenous systems.

Furthermore, covalent drug hydrogel conjugation represents a robust method for ex-tending drug retention. By linking therapeutic agents to the hydrogel backbone via cleavable chemical bonds, these systems require specific biochemical triggers (e.g., enzymatic activity or chemical stimuli) for drug activation. This adds a layer of precision to the re-lease profile, which can be tailored to disease-specific environments.

Self-healing hydrogel networks are also emerging as a transformative approach. These hydrogels can autonomously reassemble their polymeric structures following mechanical or chemical disruption, thereby preserving structural integrity and sustained drug release even under dynamic biological conditions.

Finally, the integration of advanced nanomaterials and biomimetic design principles is rapidly expanding the functional landscape of theranostic hydrogels. Incorporating engineered nanoparticles, such as biocompatible quantum dots or targeted imaging agents, not only enhances imaging contrast and therapeutic precision but also enables real-time monitoring of treatment efficacy. These multifunctional platforms support adaptive therapy based on tumor-specific responses, advancing the clinical potential of hydrogel systems in the era of personalized medicine [179,180,181].

#### 7.2.2. Integration with Advanced Technologies (AI-Driven Drug Delivery, Nanotechnology, and 3D Printing)

The convergence of multifunctional hydrogel technologies with cutting edge computational and fabrication tools holds substantial promise for clinical translation and precision medicine. Among these, AI and machine learning are emerging as powerful tools for optimizing hydrogel design and therapeutic performance. AI-based algorithms can predict drug release kinetics, model polymer degradation profiles, and identify optimal hydrogel compositions based on preclinical performance data. Furthermore, these systems facilitate the high-throughput screening of hydrogel formulations by simulating polymer drug and hydrogel tissue interactions in silico. Critically, AI can also support the personalization of hydrogel therapies by integrating patient specific parameters, such as tumor heterogeneity, gene expression profiles, and real-time imaging data, thereby enabling the development of tailored treatment regimens with enhanced efficacy and safety [182,183,184,185].

Integration with nanotechnology further enhances hydrogel functionality. Multifunctional nanomaterials embedded within hydrogels enable synchronized drug delivery, responsive behavior to environmental stimuli, and advanced imaging capabilities, thus amplifying their theranostic value. These platforms allow for simultaneous tumor targeting, real-time visualization, and feedback-regulated therapeutic activation—all critical features for adaptive cancer treatment.

Equally transformative is the application of 3D bioprinting technologies to hydrogel fabrication. This technique enables spatially resolved patterning of therapeutic agents, imaging contrast materials, and structural domains within a single hydrogel matrix. Bioprinted hydrogels can be anatomically matched to individual tumor morphologies, improving therapeutic localization and reducing off-target exposure. Additionally, 3D bioprinting facilitates improved manufacturing scalability and reproducibility by standardizing the spatial distribution and structural integrity of hydrogel-based implants.

Recent studies have demonstrated the clinical relevance of this approach. For example, a 3D bioprinted glioblastoma model using patient-derived glioma stem cells embedded in brain ECM-based bioinks enabled the assessment of drug responses to temozolomide, accurately reflecting tumor-specific therapeutic outcomes. Similarly, breast cancer models incorporating bioprinted co-cultures of tumor and stromal cells within alginate–gelatin hydrogels have successfully recapitulated tumor heterogeneity and cellular migration behaviors under chemical gradients. These models provide valuable platforms for real-time drug screening, tumor behavior analysis, and the development of precision therapies [186,187,188].

These systems have also been successfully used to fabricate implantable scaffolds for localized chemotherapy, phototherapy, and immunomodulation at resected tumor margins, demonstrating their potential for clinical application [189,190].

Incorporating these advanced technologies not only accelerates the design and optimization of multifunctional hydrogels but also strengthens their translational potential by enabling personalized, scalable, and precision-guided cancer therapy platforms.

#### 7.2.3. Personalized and Precision Medicine Approaches

Advancing personalized medicine strategies using multifunctional hydrogels represents a transformative future direction in cancer therapy. Given the inherent heterogeneity of cancers, treatments precisely tailored to patient-specific genetic profiles, tumor subtypes, or microenvironmental characteristics are essential for maximizing therapeutic success. Multifunctional hydrogels capable of dynamically responding to specific biomarkers or tumor-specific stimuli can achieve unprecedented therapeutic precision, significantly improving clinical outcomes and minimizing adverse effects [191,192].

Additionally, developing multifunctional hydrogels that are simultaneously responsive to multiple physiological and external stimuli can facilitate highly precise spatiotemporal control over therapeutic agent delivery. Coupled with integrated real-time diagnostic imaging capabilities, such advanced hydrogel systems can provide immediate clinical feedback on therapeutic efficacy, enabling prompt adjustments to treatment protocols and substantially enhancing patient prognosis [193,194,195,196].

In summary, overcoming current challenges through interdisciplinary collaboration among material scientists, biomedical researchers, clinicians, regulatory authorities, and industry partners will accelerate the successful clinical translation of multifunctional hydrogels. By leveraging recent technological advancements and personalized medicine approaches, multifunctional hydrogels hold exceptional promise as transformative tools in cancer diagnosis and therapy [197].

#### 7.2.4. Ethical and Socioeconomic Considerations

The clinical application of multifunctional hydrogels raises several ethical and socioeconomic considerations that must be addressed for equitable implementation. One key concern is the high cost associated with advanced hydrogel design, particularly when incorporating nanomaterials, bioresponsive components, or patient-specific features. These factors can limit accessibility, especially in low-resource healthcare settings. Moreover, the long-term biocompatibility of embedded nanostructures or responsive agents remains a topic of active investigation, raising ethical questions about patient safety and informed consent. Regulatory disparities between regions may further impact global accessibility. To address these challenges, future development should prioritize scalable, cost-effective production strategies and transparent safety reporting. Policies ensuring fair access, especially for underrepresented or economically disadvantaged populations, are essential to ensure that the benefits of hydrogel-based cancer therapies are universally distributed.

## 8. Conclusions

Multifunctional hydrogels represent a promising and innovative solution to overcome the significant limitations associated with conventional cancer diagnostic and therapeutic approaches. Their intrinsic biocompatibility, biodegradability, stimuli-responsive properties, and capacity to integrate advanced diagnostic imaging modalities enable simultaneous cancer diagnosis and precise, targeted therapeutic intervention. By dynamically responding to internal biological stimuli (such as pH, enzymes, or hypoxia) and external triggers (including temperature, magnetic fields, or near-infrared irradiation), multifunctional hydrogels facilitate localized, sustained drug release directly at tumor sites, significantly minimizing systemic toxicity and enhancing patient outcomes.

Integrating multifunctional hydrogels with diverse therapeutic modalities—including chemotherapy, photothermal therapy, photodynamic therapy, immunotherapy, and combination strategies—markedly improves therapeutic precision, efficacy, and patient tolerance. Injectable and in situ-forming hydrogels offer minimally invasive, patient-friendly treatment options which are particularly beneficial for postoperative cancer management, effectively reducing recurrence risk and enhancing recovery.

Despite these substantial advancements, persistent challenges remain, notably burst drug release, hydrogel stability, reproducibility in manufacturing, stringent regulatory requirements, and scalability concerns. Addressing these issues necessitates continued interdisciplinary research, innovative materials development, the integration of AI-driven personalized therapeutic systems, nanotechnological advancements, and sophisticated manufacturing techniques such as 3D bioprinting.

In conclusion, multifunctional hydrogels possess substantial transformative potential as advanced platforms in cancer management, paving the way toward safer, more effective, personalized oncology treatments.

## Figures and Tables

**Table 1:** Materials, properties, and applications of natural, synthetic, and hybrid hydrogels.

Type	Materials	Key Features	Applications	References
Natural hydrogel	Alginate, Hyaluronic Acid, Collagen, Chitosan	Biocompatibility, biodegradability, bioactivity	Drug delivery, tumor targeting	[54,55,56,57,58,59,60]
Synthetic hydrogel	PEG, PLGA, PVA, PNIPAm	Controlled mechanical strength, stimuli-responsiveness, customizable degradation rate	Controlled chemotherapy, localized immunotherapy, photothermal therapy	[61,62,63,64,65,66,67,68,69]
Hybrid hydrogel	PEG-Collagen, Chitosan-PLGA, Alginate-PEG	Optimized mechanical and biological characteristics, enhanced stimuli responsiveness	Advanced targeted delivery, precision oncology, prevention of metastasis	[70,71,72]

**Table 2:** Stimuli-responsive hydrogels for cancer treatment.

Type	Materials	Key Features	Applications	References
Stimuli-responsive hydrogel	pH-sensitive, Thermoresponsive, Magnetic-responsive, NIR-responsive hydrogels	Dynamic response to tumor microenvironment, controlled drug release, enhanced precision targeting	Localized chemotherapy, photothermal/photodynamic therapy, imaging-guided therapy	[78,79,80,81,82,83,84,85,86,87,88,89,90,91,92]

**Table 4:** Therapeutic modalities using multifunctional hydrogels for cancer treatment.

Therapuetic Modality	Cancers	Hydrogel Types	Activations	References
Chemotherapy	Breast, Lung, Ovarian	Doxorubicin, Paclitaxel-loaded hydrogels	Passive diffusion, biodegradation	[130,131,132,133,134,135]
PTT	Skin, Breast, Liver	Gold nanoparticles, Polydopamine-based hydrogels	NIR irradiation	[136,137,138,139,140]
PDT	Skin, Oral cavity	Photosensitizers (Chlorin e6, porphyrins)	Light irradiation	[141,142,143,144,145]
Immunotherapy	Melanoma, Lung	Hydrogels with checkpoint inhibitors, cytokines	Biological interaction, controlled release	[146,147,148,149,150,151,152,153,154]
Synergistic Chemo-Photothermal Therapy	Breast, Liver, Pancreatic	Hydrogels with chemotherapeutics + photothermal agents	NIR irradiation, biodegradation	[136,137]
Combined Immunotherapy and Phototherapy	Melanoma, Lung cancers	Immunotherapeutic agents + photosensitizers or photothermal agents	NIR irradiation, biodegradation, immune activation	[144,150]

## Data Availability

No new data were created or analyzed in this study. Data sharing is not applicable to this article.

## References

[1] Ferlay J., Colombet M., Soerjomataram I., Parkin D.M., Pineros M., Znaor A., Bray F. (2021). Cancer statistics for the year 2020: An overview. Int. J. Cancer.

[2] Miller K.D., Nogueira L., Devasia T., Mariotto A.B., Yabroff K.R., Jemal A., Kramer J., Siegel R.L. (2022). Cancer treatment and survivorship statistics. CA Cancer J. Clin..

[3] Zahedifard Z., Mahmoodi S., Ghasemian A. (2025). Genetically Engineered Bacteria as a Promising Therapeutic Strategy Against Cancer: A Comprehensive Review. Biotechnol. Appl. Biochem..

[4] Khan S.U., Fatima K., Aisha S., Malik F. (2024). Unveiling the mechanisms and challenges of cancer drug resistance. Cell Commun. Signal..

[5] Eslami M., Memarsadeghi O., Davarpanah A., Arti A., Nayernia K., Behnam B. (2024). Overcoming Chemotherapy Resistance in Metastatic Cancer: A Comprehensive Review. Biomedicines.

[6] Wang K., Tepper J.E. (2021). Radiation therapy-associated toxicity: Etiology, management, and prevention. CA Cancer J. Clin..

[7] Mahvi D.A., Liu R., Grinstaff M.W., Colson Y.L., Raut C.P. (2018). Local Cancer Recurrence: The Realities, Challenges, and Opportunities for New Therapies. CA Cancer J. Clin..

[8] Chargari C., Rassy E., Helissey C., Achkar S., Francois S., Deutsch E. (2023). Impact of radiation therapy on healthy tissues. Int. Rev. Cell Mol. Biol..

[9] Kaur P., Asea A. (2012). Radiation-induced effects and the immune system in cancer. Front. Oncol..

[10] Tohme S., Simmons R.L., Tsung A. (2017). Surgery for Cancer: A Trigger for Metastases. Cancer Res..

[11] Van Meir H., Nout R.A., Welters M.J., Loof N.M., de Kam M.L., van Ham J.J., Samuels S., Kenter G.G., Cohen A.F., Melief C.J. (2017). Impact of (chemo)radiotherapy on immune cell composition and function in cervical cancer patients. Oncoimmunology.

[12] Mohan G., Ayisha Hamna T.P., Jijo A.J., Saradha Devi K.M., Narayanasamy A., Vellingiri B. (2019). Recent advances in radiotherapy and its associated side effects in cancer—A review. J. Basic. Appl. Zool..

[13] Brook I. (2020). Late side effects of radiation treatment for head and neck cancer. Radiat. Oncol. J..

[14] Lee K.K., Lee J.H., Lee S.C., Lee C.S. (2022). MnCO_3_-mineralized polydopamine nanoparticles as an activatable theranostic agent for dual-modality imaging-guided photothermal therapy of cancers. Theranostics.

[15] Ryu J.H., Koo H., Sun I.C., Yuk S.H., Choi K., Kim K., Kwon I.C. (2012). Tumor-targeting multi-functional nanoparticles for theragnosis: New paradigm for cancer therapy. Adv. Drug Deliv. Rev..

[16] Dessale M., Mengistu G., Mengist H.M. (2022). Nanotechnology: A Promising Approach for Cancer Diagnosis, Therapeutics and Theragnosis. Int. J. Nanomed..

[17] Annigeri N.C., Mohan R., Vijayakumar D.B., Lingaraj A.J. (2024). Theragnosis using fluorescence: A review. J. Adv. Dent. Pract. Res..

[18] Ma Q., Li Q., Cai X., Zhou P., Wu Z., Wang B., Ma W., Fu S. (2022). Injectable hydrogels as drug delivery platform for in-situ treatment of malignant tumor. J. Drug Deliv. Sci. Technol..

[19] Mohaghegh N., Ahari A., Zehtabi F., Buttles C., Davani S., Hoang H., Tseng K., Zamanian B., Khosravi S., Daniali A. (2023). Injectable hydrogels for personalized cancer immunotherapies. Acta Biomater..

[20] Zang C., Tian Y., Tang Y., Tang M., Yang D., Chen F., Ghaffarlou M., Tu Y., Ashrafizadeh M., Li Y. (2024). Hydrogel-based platforms for site-specific doxorubicin release in cancer therapy. J. Transl. Med..

[21] Zhong Z., Gan L., Feng Z., Wang W., Pan X., Wu C., Huang Y. (2024). Hydrogel local drug delivery systems for postsurgical management of tumors: Status Quo and perspectives. Mater. Today Bio.

[22] Li X., Xu X., Xu M., Geng Z., Ji P., Liu Y. (2023). Hydrogel systems for targeted cancer therapy. Front. Bioeng. Biotechnol..

[23] Gan S., Wu Y., Zhang X., Zheng Z., Zhang M., Long L., Liao J., Chen W. (2023). Recent Advances in Hydrogel-Based Phototherapy for Tumor Treatment. Gels.

[24] Liu C., Liao Y., Liu L., Xie L., Liu J., Zhang Y., Li Y. (2023). Application of injectable hydrogels in cancer immunotherapy. Front. Bioeng. Biotechnol..

[25] Zhu H., Sun H., Dai J., Hao J., Zhou B. (2024). Chitosan-based hydrogels in cancer therapy: Drug and gene delivery, stimuli-responsive carriers, phototherapy and immunotherapy. Int. J. Biol. Macromol..

[26] Lima-Sousa R., Alves C.G., Melo B.L., Costa F.J.P., Nave M., Moreira A.F., Mendonca A.G., Correia I.J., de Melo-Diogo D. (2023). Injectable hydrogels for the delivery of nanomaterials for cancer combinatorial photothermal therapy. Biomater. Sci..

[27] Dong Y.C., Bouche M., Uman S., Burdick J.A., Cormode D.P. (2021). Detecting and Monitoring Hydrogels with Medical Imaging. ACS Biomater. Sci. Eng..

[28] Fu L., Ke H.T. (2016). Nanomaterials incorporated ultrasound contrast agents for cancer theranostics. Cancer Biol. Med..

[29] Mieszawska A.J., Mulder W.J., Fayad Z.A., Cormode D.P. (2013). Multifunctional gold nanoparticles for diagnosis and therapy of disease. Mol. Pharm..

[30] Priester M.I., Ten Hagen T.L.M. (2023). Image-guided drug delivery in nanosystem-based cancer therapies. Adv. Drug Deliv. Rev..

[31] Xu X., Liu Y., Liu Y., Yu Y., Yang M., Lu L., Chan L., Liu B. (2024). Functional hydrogels for hepatocellular carcinoma: Therapy, imaging, and in vitro model. J. Nanobiotechnol..

[32] Zhang Y., Tian S., Huang L., Li Y., Lu Y., Li H., Chen G., Meng F., Liu G.L., Yang X. (2022). Reactive oxygen species-responsive and Raman-traceable hydrogel combining photodynamic and immune therapy for postsurgical cancer treatment. Nat. Commun..

[33] Walker E., Linders D.G.J., Abenojar E., Wang X., Hazelbag H.M., Straver M.E., Bijlstra O.D., March T.L., Vahrmeijer A.L., Exner A. (2022). Formulation of a Thermosensitive Imaging Hydrogel for Topical Application and Rapid Visualization of Tumor Margins in the Surgical Cavity. Cancers.

[34] Wang H., Mao D., Wang Y., Wang K., Yi X., Kong D., Yang Z., Liu Q., Ding D. (2015). Biocompatible fluorescent supramolecular nanofibrous hydrogel for long-term cell tracking and tumor imaging applications. Sci. Rep..

[35] Nicolson F., Andreiuk B., Lee E., O’Donnell B., Whitley A., Riepl N., Burkhart D.L., Cameron A., Protti A., Rudder S. (2024). In vivo imaging using surface enhanced spatially offset raman spectroscopy (SESORS): Balancing sampling frequency to improve overall image acquisition. Npj Imaging.

[36] Zhang Y., Wu B.M. (2023). Current Advances in Stimuli-Responsive Hydrogels as Smart Drug Delivery Carriers. Gels.

[37] Wells C.M., Harris M., Choi L., Murali V.P., Guerra F.D., Jennings J.A. (2019). Stimuli-Responsive Drug Release from Smart Polymers. J. Funct. Biomater..

[38] Liu Y., Ran Y., Ge Y., Raza F., Li S., Zafar H., Wu Y., Paiva-Santos A.C., Yu C., Sun M. (2022). pH-Sensitive Peptide Hydrogels as a Combination Drug Delivery System for Cancer Treatment. Pharmaceutics.

[39] Li M., Zhao G., Su W.K., Shuai Q. (2020). Enzyme-Responsive Nanoparticles for Anti-tumor Drug Delivery. Front. Chem..

[40] Feng H., Chu D., Yang F., Li Z., Fan B., Jin L., Li J. (2020). Hypoxia-Responsive Polymeric Micelles for Enhancing Cancer Treatment. Front. Chem..

[41] Abed H.F., Abuwatfa W.H., Husseini G.A. (2022). Redox-Responsive Drug Delivery Systems: A Chemical Perspective. Nanomaterials.

[42] Tanga S., Aucamp M., Ramburrun P. (2023). Injectable Thermoresponsive Hydrogels for Cancer Therapy: Challenges and Prospects. Gels.

[43] Londhe P.V., Londhe M.V., Salunkhe A.B., Laha S.S., Mefford O.T., Thorat N.D., Khot V.M. (2025). Magnetic hydrogel (MagGel): An evolutionary pedestal for anticancer therapy. Coord. Chem. Rev..

[44] Sun Y., Chen L.G., Fan X.M., Pang J.L. (2022). Ultrasound Responsive Smart Implantable Hydrogels for Targeted Delivery of Drugs: Reviewing Current Practices. Int. J. Nanomed..

[45] Zhao C., Pan B., Wang T., Yang H., Vance D., Li X., Zhao H., Hu X., Yang T., Chen Z. (2023). Advances in NIR-Responsive Natural Macromolecular Hydrogel Assembly Drugs for Cancer Treatment. Pharmaceutics.

[46] Xu X., Liu Y., Fu W., Yao M., Ding Z., Xuan J., Li D., Wang S., Xia Y., Cao M. (2020). Poly(N-isopropylacrylamide)-Based Thermoresponsive Composite Hydrogels for Biomedical Applications. Polymers.

[47] Gu J., Zhao G., Yu J., Xu P., Yan J., Jin Z., Chen S., Wang Y., Zhang L.W., Wang Y. (2022). Injectable pH-responsive hydrogel for combinatorial chemoimmunotherapy tailored to the tumor microenvironment. J. Nanobiotechnol..

[48] Bossmann S.H., Payne M.M., Kalita M., Bristow R.M.D., Afshar A., Perera A.S. (2022). Iron-Based Magnetic Nanosystems for Diagnostic Imaging and Drug Delivery: Towards Transformative Biomedical Applications. Pharmaceutics.

[49] Wang Z., Zhai B., Sun J., Zhang X., Zou J., Shi Y., Guo D. (2024). Recent advances of injectable in situ-forming hydrogels for preventing postoperative tumor recurrence. Drug Deliv..

[50] Feng Y., Zhang Z., Tang W., Dai Y. (2023). Gel/hydrogel-based in situ biomaterial platforms for cancer postoperative treatment and recovery. Exploration.

[51] Go K., Kim D.-M., Lee K.J. (2024). 3D printable hydrogel filament with functionalizable moiety for in-situ flow-based sensor. Macromol. Res..

[52] Bhuskute H., Shende P., Prabhakar B. (2021). 3D Printed Personalized Medicine for Cancer: Applications for Betterment of Diagnosis, Prognosis and Treatment. AAPS PharmSciTech.

[53] Ma Y., Deng B., He R., Huang P. (2024). Advancements of 3D bioprinting in regenerative medicine: Exploring cell sources for organ fabrication. Heliyon.

[54] Fahad A., Bagher F. (2024). Recent advances on chitosan/hyaluronic acid-based stimuli-responsive hydrogels and composites for cancer treatment: A comprehensive review. Int. J. Biol. Macromol..

[55] Pratikshya P., Tarun K.U., Nawaf A., Mohd S., Kavindra K.K. (2024). Alginate-Chitosan Biodegradable and Biocompatible Based Hydrogel for Breast Cancer Immunotherapy and Diagnosis: A Comprehensive Review. ACS Appl. Bio Mater..

[56] Ferreira N.N., Ferreira L.M.B., Miranda-Goncalves V., Reis R.M., Seraphim T.V., Borges J.C., Baltazar F., Gremiao M.P.D. (2017). Alginate hydrogel improves anti-angiogenic bevacizumab activity in cancer therapy. Eur. J. Pharm. Biopharm..

[57] Rezk A.I., Obiweluozor F.O., Choukrani G., Park C.H., Kim C.S. (2019). Drug release and kinetic models of anticancer drug (BTZ) from a pH-responsive alginate polydopamine hydrogel: Towards cancer chemotherapy. Int. J. Biol. Macromol..

[58] Zhang Y., Wang T., Zhuang Y., He T., Wu X., Su L., Kang J., Chang J., Wang H. (2021). Sodium Alginate Hydrogel-Mediated Cancer Immunotherapy for Postoperative In Situ Recurrence and Metastasis. ACS Biomater. Sci. Eng..

[59] Song W., Su X., Gregory D.A., Li W., Cai Z., Zhao X. (2018). Magnetic Alginate/Chitosan Nanoparticles for Targeted Delivery of Curcumin into Human Breast Cancer Cells. Nanomaterials.

[60] Shanmugapriya K., Kang H.W. (2021). Synthesis of nanohydroxyapatite/collagen-loaded fucoidan-based composite hydrogel for drug delivery to gastrointestinal cancer cells. Colloids Surf. B Biointerfaces.

[61] Zhang F., Zhang S., Lin R., Cui S., Jing X., Coseri S. (2023). Injectable multifunctional carboxymethyl chitosan/hyaluronic acid hydrogel for drug delivery systems. Int. J. Biol. Macromol..

[62] Ren Y., Zhao X., Liang X., Ma P.X., Guo B. (2017). Injectable hydrogel based on quaternized chitosan, gelatin and dopamine as localized drug delivery system to treat Parkinson’s disease. Int. J. Biol. Macromol..

[63] Wang X., Wang C., Wang X., Wang Y., Zhang Q., Cheng Y. (2017). A Polydopamine Nanoparticle-Knotted Poly(ethylene glycol) Hydrogel for On-Demand Drug Delivery and Chemo-photothermal Therapy. Chem. Mater..

[64] Liu C., Guo X., Ruan C., Hu H., Jiang B.P., Liang H., Shen X.C. (2019). An injectable thermosensitive photothermal-network hydrogel for near-infrared-triggered drug delivery and synergistic photothermal-chemotherapy. Acta Biomater..

[65] Rong X., Ji Y., Zhu X., Yang J., Qian D., Mo X., Lu Y. (2019). Neuroprotective effect of insulin-loaded chitosan nanoparticles/PLGA-PEG-PLGA hydrogel on diabetic retinopathy in rats. Int. J. Nanomed..

[66] Yang Z., Yu S., Li D., Gong Y., Zang J., Liu J., Chen X. (2018). The effect of PLGA-based hydrogel scaffold for improving the drug maximum-tolerated dose for in situ osteosarcoma treatment. Colloids Surf. B Biointerfaces.

[67] Zhai L., Shi Y., Yan Y., Lu A., Liu X., Lei L., Sun Y., Jiang L., Wang X., Qian H. (2023). Local sustained release of PD-1 monoclonal antibody and lenvatinib by thermo-sensitive hydrogel for improving tumor immunotherapy. Chin. Chem. Lett..

[68] Dhamecha D., Le D., Chakravarty T., Perera K., Dutta A., Menon J.U. (2021). Fabrication of PNIPAm-based thermoresponsive hydrogel microwell arrays for tumor spheroid formation. Mater. Sci. Eng. C Mater. Biol. Appl..

[69] Fang Y., Tan J., Lim S., Soh S. (2018). Rupturing cancer cells by the expansion of functionalized stimuli-responsive hydrogels. NPG Asia Mater..

[70] Zhang Q., Li J., Qu Q., Pan S., Yu K., Liu Y. (2024). Graphene oxide modified sodium alginate/polyethylene glycol phase change material hydrogel scaffold composite with photothermal temperature control for potential bone tissue regeneration. J. Mater. Res. Technol..

[71] Baraian A.I., Raduly L., Zanoaga O., Iacob B.C., Barbu-Tudoran L., Dinte E., Berindan-Neagoe I., Bodoki E. (2025). Targeting JAK/STAT3 in glioblastoma cells using an alginate-PNIPAm molecularly imprinted hydrogel for the sustained release of ruxolitinib. Int. J. Biol. Macromol..

[72] Nam S., Stowers R., Lou J., Xia Y., Chaudhuri O. (2019). Varying PEG density to control stress relaxation in alginate-PEG hydrogels for 3D cell culture studies. Biomaterials.

[73] Sargeant T.D., Desai A.P., Banerjee S., Agawu A., Stopek J.B. (2012). An in situ forming collagen-PEG hydrogel for tissue regeneration. Acta Biomater..

[74] Jin X., Fu Q., Gu Z., Zhang Z., Lv H. (2020). Injectable corilagin/low molecular weight chitosan/PLGA-PEG-PLGA thermosensitive hydrogels for localized cancer therapy and promoting drug infiltration by modulation of tumor microenvironment. Int. J. Pharm..

[75] Kim T.T., Malu D., He D., Hu Y., Kim J. (2025). Development of Bioorthogonally Degradable Tough Hydrogels Using Enamine N-Oxide Based Crosslinkers. Adv. Mater..

[76] Liu Z., Koseki Y., Suzuki R., Dao A.T.N., Kasai H. (2025). Sustained Drug Release from Dual-Responsive Hydrogels for Local Cancer Chemo–Photothermal Therapy. Macromol. Biosci..

[77] Tang M., Song J., Zhang S., Shu X., Liu S., Ashrafizadeh M., Ertas Y.N., Zhou Y., Lei M. (2024). Innovative theranostic hydrogels for targeted gastrointestinal cancer treatment. J. Transl. Med..

[78] Bilalis P., Skoulas D., Karatzas A., Marakis J., Stamogiannos A., Tsimblouli C., Sereti E., Stratikos E., Dimas K., Vlassopoulos D. (2018). Self-Healing pH- and Enzyme Stimuli-Responsive Hydrogels for Targeted Delivery of Gemcitabine To Treat Pancreatic Cancer. Biomacromolecules.

[79] Kozlovskaya V., Chen J., Tedjo C., Liang X., Campos-Gomez J., Oh J., Saeed M., Lungu C.T., Kharlampieva E. (2014). pH-responsive hydrogel cubes for release of doxorubicin in cancer cells. J. Mater. Chem. B.

[80] Raza F., Zhu Y., Chen L., You X., Zhang J., Khan A., Khan M.W., Hasnat M., Zafar H., Wu J. (2019). Paclitaxel-loaded pH responsive hydrogel based on self-assembled peptides for tumor targeting. Biomater. Sci..

[81] Song X., Zhang Z., Zhu J., Wen Y., Zhao F., Lei L., Phan-Thien N., Khoo B.C., Li J. (2020). Thermoresponsive Hydrogel Induced by Dual Supramolecular Assemblies and Its Controlled Release Property for Enhanced Anticancer Drug Delivery. Biomacromolecules.

[82] Luo Y., Li J., Hu Y., Gao F., Pak-Heng Leung G., Geng F., Fu C., Zhang J. (2020). Injectable thermo-responsive nano-hydrogel loading triptolide for the anti-breast cancer enhancement via localized treatment based on “two strikes” effects. Acta Pharm. Sin. B.

[83] Wang C., Zhang G., Liu G., Hu J., Liu S. (2017). Photo- and thermo-responsive multicompartment hydrogels for synergistic delivery of gemcitabine and doxorubicin. J. Control. Release.

[84] Jaiswal M.K., De M., Chou S.S., Vasavada S., Bleher R., Prasad P.V., Bahadur D., Dravid V.P. (2014). Thermoresponsive magnetic hydrogels as theranostic nanoconstructs. ACS Appl. Mater. Interfaces.

[85] Zeng N., He L., Jiang L., Shan S., Su H. (2022). Synthesis of magnetic/pH dual responsive dextran hydrogels as stimuli-sensitive drug carriers. Carbohydr. Res..

[86] Jo Y.J., Gulfam M., Jo S.H., Gal Y.S., Oh C.W., Park S.H., Lim K.T. (2022). Multi-stimuli responsive hydrogels derived from hyaluronic acid for cancer therapy application. Carbohydr. Polym..

[87] Arjama M., Mehnath S., Jeyaraj M. (2022). Self-assembled hydrogel nanocube for stimuli responsive drug delivery and tumor ablation by phototherapy against breast cancer. Int. J. Biol. Macromol..

[88] He G., Chen S., Xu Y., Miao Z., Ma Y., Qian H., Lu Y., Zha Z. (2019). Charge reversal induced colloidal hydrogel acts as a multi-stimuli responsive drug delivery platform for synergistic cancer therapy. Mater. Horiz..

[89] Wu Y., Chen F., Huang N., Li J., Wu C., Tan B., Liu Y., Li L., Yang C., Shao D. (2021). Near-infrared light-responsive hybrid hydrogels for the synergistic chemo-photothermal therapy of oral cancer. Nanoscale.

[90] Lv S.W., Liu Y., Xie M., Wang J., Yan X.W., Li Z., Dong W.G., Huang W.H. (2016). Near-Infrared Light-Responsive Hydrogel for Specific Recognition and Photothermal Site-Release of Circulating Tumor Cells. ACS Nano.

[91] Hao Y., Dong Z., Chen M., Chao Y., Liu Z., Feng L., Hao Y., Dong Z.L., Chen M.C., Chao Y. (2020). Near-infrared light and glucose dual-responsive cascading hydroxyl radical generation for in situ gelation and effective breast cancer treatment. Biomaterials.

[92] Kawamura A., Harada A., Ueno S., Miyata T. (2021). Weakly Acidic pH and Reduction Dual Stimuli-Responsive Gel Particles. Langmuir.

[93] Abourehab M.A.S., Rajendran R.R., Singh A., Pramanik S., Shrivastav P., Ansari J., Manne R., Amaral L.S., Deepak A. (2022). Alginate as a Promising Biopolymer in Drug Delivery and Wound Healing: A Review of the State-of-the-Art. Int. J. Mol. Sci..

[94] Hu W., Wang Z., Xiao Y., Zhang S., Wang J. (2019). Advances in Crosslinking Strategies of Biomedical Hydrogels. Biomater. Sci..

[95] Maitra J., Shukla V.K. (2014). Cross-linking in Hydrogels—A Review. Am. J. Polym. Sci..

[96] Rebers L., Reichsöllner R., Regett S., Tovar G.E.M., Borchers K., Baudis S., Southan A. (2021). Differentiation of Physical and Chemical Cross-Linking in Gelatin Methacryloyl Hydrogels. Sci. Rep..

[97] Nieto D., Marchal Corrales J.A., de Mora A.J., Moroni L. (2020). Fundamentals of Light-Cell–Polymer Interactions in Photo-Cross-Linking Based Bioprinting. APL Bioeng..

[98] Gaudet I.D., Shreiber D.I. (2012). Characterization of Methacrylated Type-I Collagen as a Dynamic, Photoactive Hydrogel. Biointerphases.

[99] Augustine R., Kalva S.N., Ahmad R., Zahid A.A., Hasan S., Nayeem A., McClements L., Hasan A. (2021). 3D Bioprinted Cancer Models: Revolutionizing Personalized Cancer Therapy. Transl. Oncol..

[100] Shuhendler A.J., Staruch R., Oakden W., Gordijo C.R., Rauth A.M., Stanisz G.J., Chopra R., Wu X.Y. (2012). Thermally-triggered ‘off-on-off’ response of gadolinium-hydrogel-lipid hybrid nanoparticles defines a customizable temperature window for non-invasive magnetic resonance imaging thermometry. J. Control. Release.

[101] Courant T., Roullin V.G., Cadiou C., Callewaert M., Andry M.C., Portefaix C., Hoeffel C., de Goltstein M.C., Port M., Laurent S. (2012). Hydrogels incorporating GdDOTA: Towards highly efficient dual *T*_1_/*T*_2_ MRI contrast agents. Angew. Chem. Int. Ed. Engl..

[102] Liu J., Wang K., Luan J., Wen Z., Wang L., Liu Z., Wu G., Zhuo R. (2016). Visualization of in situ hydrogels by MRI in vivo. J. Mater. Chem. B.

[103] Shazeeb M.S., Corazzini R., Konowicz P.A., Fogle R., Bangari D.S., Johnson J., Ying X., Dhal P.K. (2018). Assessment of in vivo degradation profiles of hyaluronic acid hydrogels using temporal evolution of chemical exchange saturation transfer (CEST) MRI. Biomaterials.

[104] Dorsey S.M., Haris M., Singh A., Witschey W.R.T., Rodell C.B., Kogan F., Reddy R., Burdick J.A. (2015). Visualization of Injectable Hydrogels Using Chemical Exchange Saturation Transfer MRI. ACS Biomater. Sci. Eng..

[105] Zhu W., Chu C., Kuddannaya S., Yuan Y., Walczak P., Singh A., Song X., Bulte J.W.M. (2019). In Vivo Imaging of Composite Hydrogel Scaffold Degradation Using CEST MRI and Two-Color NIR Imaging. Adv. Funct. Mater..

[106] Kim S.D., Park K., Lee S., Kum J., Kim Y., An S., Kim H., Shin M., Son D. (2023). Injectable and tissue-conformable conductive hydrogel for MRI-compatible brain-interfacing electrodes. Soft Sci..

[107] Bermejo-Velasco D., Dou W., Heerschap A., Ossipov D., Hilborn J. (2018). Injectable hyaluronic acid hydrogels with the capacity for magnetic resonance imaging. Carbohydr. Polym..

[108] Chen X., Zhang J., Wu K., Wu X., Tang J., Cui S., Cao D., Liu R., Peng C., Yu L. (2020). Visualizing the In Vivo Evolution of an Injectable and Thermosensitive Hydrogel Using Tri-Modal Bioimaging. Small Methods.

[109] Zhang J., Jin J., Wan J., Jiang S., Wu Y., Wang W., Gong X., Wang H. (2021). Quantum dots-based hydrogels for sensing applications. Chem. Eng. J..

[110] Park G.K., Kim S.H., Kim K., Das P., Kim B.G., Kashiwagi S., Choi H.S., Hwang N.S. (2019). Dual-Channel Fluorescence Imaging of Hydrogel Degradation and Tissue Regeneration in the Brain. Theranostics.

[111] Wang L., Li B., Xu F., Li Y., Xu Z., Wei D., Feng Y., Wang Y., Jia D., Zhou Y. (2017). Visual in vivo degradation of injectable hydrogel by real-time and non-invasive tracking using carbon nanodots as fluorescent indicator. Biomaterials.

[112] Lee S.S., Kim H., Sohn D.K., Eom J.B., Seo Y.S., Yoon H.M., Choi Y. (2020). Indocyanine green-loaded injectable alginate hydrogel as a marker for precision cancer surgery. Quant. Imaging Med. Surg..

[113] Sachdev A., Matai I., Gopinath P. (2016). Carbon dots incorporated polymeric hydrogels as multifunctional platform for imaging and induction of apoptosis in lung cancer cells. Colloids Surf. B Biointerfaces.

[114] Mohammadi S., Mohammadi S., Salimi A. (2021). A 3D hydrogel based on chitosan and carbon dots for sensitive fluorescence detection of microRNA-21 in breast cancer cells. Talanta.

[115] Dong X., Liang J., Yang A., Qian Z., Kong D., Lv F. (2019). Fluorescence imaging guided CpG nanoparticles-loaded IR820-hydrogel for synergistic photothermal immunotherapy. Biomaterials.

[116] Back W., Rho J., Kim K., Yong H.S., Jeon O.H., Choi B.H., Kim H.K., Park J.H. (2024). An injectable fluorescent and iodinated hydrogel for preoperative localization and dual image-guided surgery of pulmonary nodules. Biomater. Sci..

[117] Patrick P.S., Bear J.C., Fitzke H.E., Zaw-Thin M., Parkin I.P., Lythgoe M.F., Kalber T.L., Stuckey D.J. (2020). Radio-metal cross-linking of alginate hydrogels for non-invasive in vivo imaging. Biomaterials.

[118] Gu X., Shu Z., Zheng X., Wei S., Ma M., He H., Shi Y., Gong X., Chen S., Wang X. (2023). A novel CT-responsive hydrogel for the construction of an organ simulation phantom for the repeatability and stability study of radiomic features. J. Mater. Chem. B.

[119] Dong Y.C., Kumar A., Rosario-Berrios D.N., Si-Mohamed S., Hsu J.C., Nieves L.M., Douek P., Noel P.B., Cormode D.P. (2022). Ytterbium Nanoparticle Contrast Agents for Conventional and Spectral Photon-Counting CT and Their Applications for Hydrogel Imaging. ACS Appl. Mater. Interfaces.

[120] Exner A.A., Kolios M.C. (2021). Bursting Microbubbles: How Nanobubble Contrast Agents Can Enable the Future of Medical Ultrasound Molecular Imaging and Image-Guided Therapy. Curr. Opin. Colloid Interface Sci..

[121] Helfield B., Zou Y., Matsuura N. (2021). Acoustically-Stimulated Nanobubbles: Opportunities in Medical Ultrasound Imaging and Therapy. Front. Phys..

[122] Zhu W., Zhou Z., Huang Y., Liu H., He N., Zhu X., Han X., Zhou D., Duan X., Chen X. (2023). A versatile 3D-printable hydrogel for antichondrosarcoma, antibacterial, and tissue repair. J. Mater. Sci. Technol..

[123] Zheng N., Fitzpatrick V., Cheng R., Shi L., Kaplan D.L., Yang C. (2022). Photoacoustic Carbon Nanotubes Embedded Silk Scaffolds for Neural Stimulation and Regeneration. ACS Nano.

[124] Jin R., Yang X., Zhao D., Hou X., Li C., Song X., Chen W., Wang Q., Zhao Y., Liu B. (2019). An injectable hybrid hydrogel based on a genetically engineered polypeptide for second near-infrared fluorescence/photoacoustic imaging-monitored sustained chemo-photothermal therapy. Nanoscale.

[125] Xiao Y., Pandey K., Nicolas-Boluda A., Onidas D., Nizard P., Carn F., Lucas T., Gateau J., Martin-Molina A., Quesada-Perez M. (2022). Synergic Thermo- and pH-Sensitive Hybrid Microgels Loaded with Fluorescent Dyes and Ultrasmall Gold Nanoparticles for Photoacoustic Imaging and Photothermal Therapy. ACS Appl. Mater. Interfaces.

[126] Jin R.-M., Yao M.-H., Yang J., Zhao D.-H., Zhao Y.-D., Liu B. (2017). One-Step in Situ Synthesis of Polypeptide–Gold Nanoparticles Hybrid Nanogels and Their Application in Targeted Photoacoustic Imaging. ACS Sustain. Chem. Eng..

[127] Kotturi D., Paterson S., McShane M. (2021). Comparison of SERS pH probe responses after microencapsulation within hydrogel matrices. J. Biomed. Opt..

[128] Wang W., Vikesland P.J. (2023). SERS-Active Printable Hydrogel for 3D Cell Culture and Imaging. Anal. Chem..

[129] Kim D., Gwon G., Lee G., Jeon Y., Kim U.J., Alothman Z.A., You J. (2021). Surface-enhanced Raman scattering-active AuNR array cellulose films for multi-hazard detection. J. Hazard. Mater..

[130] Gallo E., Diaferia C., Rosa E., Smaldone G., Morelli G., Accardo A. (2021). Peptide-Based Hydrogels and Nanogels for Delivery of Doxorubicin. Int. J. Nanomed..

[131] Hyun H., Yoo Y.B., Kim S.Y., Ko H.S., Chun H.J., Yang D.H. (2019). Hydrogel-Mediated DOX⋅HCl/PTX Delivery System for Breast Cancer Therapy. Int. J. Mol. Sci..

[132] Sheu M.T., Jhan H.J., Su C.Y., Chen L.C., Chang C.E., Liu D.Z., Ho H.O. (2016). Codelivery of doxorubicin-containing thermosensitive hydrogels incorporated with docetaxel-loaded mixed micelles enhances local cancer therapy. Colloids Surf. B Biointerfaces.

[133] Wu Z., Zou X., Yang L., Lin S., Fan J., Yang B., Sun X., Wan Q., Chen Y., Fu S. (2014). Thermosensitive hydrogel used in dual drug delivery system with paclitaxel-loaded micelles for in situ treatment of lung cancer. Colloids Surf. B Biointerfaces.

[134] Shen W., Chen X., Luan J., Wang D., Yu L., Ding J. (2017). Sustained Codelivery of Cisplatin and Paclitaxel via an Injectable Prodrug Hydrogel for Ovarian Cancer Treatment. ACS Appl. Mater. Interfaces.

[135] Nieto C., Vega M.A., Rodriguez V., Perez-Esteban P., Martin Del Valle E.M. (2022). Biodegradable gellan gum hydrogels loaded with paclitaxel for HER2+ breast cancer local therapy. Carbohydr. Polym..

[136] Xu X., Huang Z., Huang Z., Zhang X., He S., Sun X., Shen Y., Yan M., Zhao C. (2017). Injectable, NIR/pH-Responsive Nanocomposite Hydrogel as Long-Acting Implant for Chemophotothermal Synergistic Cancer Therapy. ACS Appl. Mater. Interfaces.

[137] Xie W., Gao Q., Guo Z., Wang D., Gao F., Wang X., Wei Y., Zhao L. (2017). Injectable and Self-Healing Thermosensitive Magnetic Hydrogel for Asynchronous Control Release of Doxorubicin and Docetaxel to Treat Triple-Negative Breast Cancer. ACS Appl. Mater. Interfaces.

[138] Fong Y.T., Chen C.H., Chen J.P. (2017). Intratumoral Delivery of Doxorubicin on Folate-Conjugated Graphene Oxide by In-Situ Forming Thermo-Sensitive Hydrogel for Breast Cancer Therapy. Nanomaterials.

[139] Liu M., Huang P., Wang W., Feng Z., Zhang J., Deng L., Dong A. (2019). An injectable nanocomposite hydrogel co-constructed with gold nanorods and paclitaxel-loaded nanoparticles for local chemo-photothermal synergetic cancer therapy. J. Mater. Chem. B.

[140] Zhang H., Zhu X., Ji Y., Jiao X., Chen Q., Hou L., Zhang H., Zhang Z. (2015). Near-infrared-triggered in situ hybrid hydrogel system for synergistic cancer therapy. J. Mater. Chem. B.

[141] Xia L.Y., Zhang X., Cao M., Chen Z., Wu F.G. (2017). Enhanced Fluorescence Emission and Singlet Oxygen Generation of Photosensitizers Embedded in Injectable Hydrogels for Imaging-Guided Photodynamic Cancer Therapy. Biomacromolecules.

[142] Yue J., Miao P., Li L., Yan R., Dong W.F., Mei Q. (2022). Injectable Carbon Dots-Based Hydrogel for Combined Photothermal Therapy and Photodynamic Therapy of Cancer. ACS Appl. Mater. Interfaces.

[143] Chen T., Yao T., Peng H., Whittaker A.K., Li Y., Zhu S., Wang Z. (2021). An Injectable Hydrogel for Simultaneous Photothermal Therapy and Photodynamic Therapy with Ultrahigh Efficiency Based on Carbon Dots and Modified Cellulose Nanocrystals. Adv. Funct. Mater..

[144] Leung B., Dharmaratne P., Yan W., Chan B.C.L., Lau C.B.S., Fung K.P., Ip M., Leung S.S.Y. (2020). Development of thermosensitive hydrogel containing methylene blue for topical antimicrobial photodynamic therapy. J. Photochem. Photobiol. B.

[145] Karuppusamy S., Hyejin K., Kang H.W. (2019). Nanoengineered chlorin e6 conjugated with hydrogel for photodynamic therapy on cancer. Colloids Surf. B Biointerfaces.

[146] Song H., Yang P., Huang P., Zhang C., Kong D., Wang W. (2019). Injectable polypeptide hydrogel-based co-delivery of vaccine and immune checkpoint inhibitors improves tumor immunotherapy. Theranostics.

[147] Zhang H., Zhang J., Liu Y., Jiang Y., Li Z. (2021). Molecular Targeted Agent and Immune Checkpoint Inhibitor Co-Loaded Thermosensitive Hydrogel for Synergistic Therapy of Rectal Cancer. Front. Pharmacol..

[148] Wang F., Su H., Xu D., Monroe M.K., Anderson C.F., Zhang W., Oh R., Wang Z., Sun X., Wang H. (2021). Therapeutic supramolecular tubustecan hydrogel combined with checkpoint inhibitor elicits immunity to combat cancer. Biomaterials.

[149] Chen Z., Rong Y., Ding J., Cheng X., Chen X., He C. (2023). Injectable Polypeptide Hydrogel Depots Containing Dual Immune Checkpoint Inhibitors and Doxorubicin for Improved Tumor Immunotherapy and Post-Surgical Tumor Treatment. Pharmaceutics.

[150] Hao Y., Chung C.K., Gu Z., Schomann T., Dong X., Veld R., Camps M.G.M., Ten Dijke P., Ossendorp F.A., Cruz L.J. (2022). Combinatorial therapeutic approaches of photodynamic therapy and immune checkpoint blockade for colon cancer treatment. Mol. Biomed..

[151] Du Y.N., Wei Q., Zhao L.J., Fan C.Q., Guo L.R., Ye J.F., Li Y. (2022). Hydrogel-based co-delivery of CIK cells and oncolytic adenovirus armed with IL12 and IL15 for cancer immunotherapy. Biomed. Pharmacother..

[152] Xiong Z., Sun L., Yang H., Xiao Z., Deng Z., Li Q., Wang C., Shen F., Liu Z. (2022). Ni-Alginate Hydrogel Microspheres with Sustained Interleukin 2 Release to Boost Cytokine-Based Cancer Immunotherapy. Adv. Funct. Mater..

[153] Lv Q., He C., Quan F., Yu S., Chen X. (2018). DOX/IL-2/IFN-gamma co-loaded thermo-sensitive polypeptide hydrogel for efficient melanoma treatment. Bioact. Mater..

[154] Sun L., Shen F., Tian L., Tao H., Xiong Z., Xu J., Liu Z. (2021). ATP-Responsive Smart Hydrogel Releasing Immune Adjuvant Synchronized with Repeated Chemotherapy or Radiotherapy to Boost Antitumor Immunity. Adv. Mater..

[155] Fathi M., Majidi S., Zangabad P.S., Barar J., Erfan-Niya H., Omidi Y. (2018). Chitosan-based multifunctional nanomedicines and theranostics for targeted therapy of cancer. Med. Res. Rev..

[156] Wang F., Chen J., Liu J., Zeng H. (2021). Cancer theranostic platforms based on injectable polymer hydrogels. Biomater. Sci..

[157] Kim J., Choi Y., Kim D.H., Yoon H.Y., Kim K. (2022). Injectable Hydrogel-Based Combination Cancer Immunotherapy for Overcoming Localized Therapeutic Efficacy. Pharmaceutics.

[158] Zhao L., Zhu L., Liu F., Liu C., Shan D., Wang Q., Zhang C., Li J., Liu J., Qu X. (2011). pH triggered injectable amphiphilic hydrogel containing doxorubicin and paclitaxel. Int. J. Pharm..

[159] Zhao Z., Li Q., Qin X., Zhang M., Du Q., Luan Y. (2022). An Injectable Hydrogel Reshaping Adenosinergic Axis for Cancer Therapy. Adv. Funct. Mater..

[160] Kumar S., Bajaj A. (2020). Advances in self-assembled injectable hydrogels for cancer therapy. Biomater. Sci..

[161] Gil M.S., Thambi T., Phan V.H.G., Kim S.H., Lee D.S. (2017). Injectable hydrogel-incorporated cancer cell-specific cisplatin releasing nanogels for targeted drug delivery. J. Mater. Chem. B.

[162] Lee K.K., Lee S.C., Kim H., Lee C.-S. (2022). Polydopamine Nanoparticle-Incorporated Fluorescent Hydrogel for Fluorescence Imaging-Guided Photothermal Therapy of Cancers. BioChip J..

[163] Jia Y.P., Shi K., Yang F., Liao J.F., Han R.X., Yuan L.P., Hao Y., Pan M., Xiao Y., Qian Z.Y. (2020). Multifunctional Nanoparticle Loaded Injectable Thermoresponsive Hydrogel as NIR Controlled Release Platform for Local Photothermal Immunotherapy to Prevent Breast Cancer Postoperative Recurrence and Metastases. Adv. Funct. Mater..

[164] Mi D., Li J., Wang R., Li Y., Zou L., Sun C., Yan S., Yang H., Zhao M., Shi S. (2023). Postsurgical wound management and prevention of triple-negative breast cancer recurrence with a pryoptosis-inducing, photopolymerizable hydrogel. J. Control. Release.

[165] Wang H., Jin Y., Tan Y., Zhu H., Huo W., Niu P., Li Z., Zhang J., Liang X.J., Yang X. (2021). Photo-responsive hydrogel facilitates nutrition deprivation by an ambidextrous approach for preventing cancer recurrence and metastasis. Biomaterials.

[166] Li J., Chen Q., Li S., Zeng X., Qin J., Li X., Chen Z., Zheng W., Zhao Y., Huang Z. (2023). An adhesive hydrogel implant combining chemotherapy and tumor microenvironment remodeling for preventing postoperative recurrence and metastasis of breast cancer. Chem. Eng. J..

[167] Zhuang B., Chen T., Xiao Z., Jin Y. (2020). Drug-loaded implantable surgical cavity-adaptive hydrogels for prevention of local tumor recurrence. Int. J. Pharm..

[168] Piantanida E., Alonci G., Bertucci A., De Cola L. (2019). Design of Nanocomposite Injectable Hydrogels for Minimally Invasive Surgery. Acc. Chem. Res..

[169] Tan B., Huang L., Wu Y., Liao J. (2021). Advances and trends of hydrogel therapy platform in localized tumor treatment: A review. J. Biomed. Mater. Res. A.

[170] Zhao F., Yao D., Guo R., Deng L., Dong A., Zhang J. (2015). Composites of Polymer Hydrogels and Nanoparticulate Systems for Biomedical and Pharmaceutical Applications. Nanomaterials.

[171] Hoare T.R., Kohane D.S. (2008). Hydrogels in drug delivery: Progress and challenges. Polymer.

[172] Zhang J., Lin X., Liu J., Zhao J., Dong H., Deng L., Liu J., Dong A. (2013). Sequential thermo-induced self-gelation and acid-triggered self-release process of drug-conjugated nanoparticles: A strategy for the sustained and controlled drug delivery to tumors. J. Mater. Chem. B.

[173] Laquerbe S., Es Sayed J., Lorthioir C., Meyer C., Narita T., Ducouret G., Perrin P., Sanson N. (2023). Supramolecular Crosslinked Hydrogels: Similarities and Differences with Chemically Crosslinked Hydrogels. Macromolecules.

[174] Zhang Z., Li T., Liu Y., Shang F., Chen B., Hu Y., Wang S., Guo Z. (2017). Supramolecular hydrogel of poly(vinyl alcohol)/chitosan: A dual cross-link design. Adv. Polym. Technol..

[175] Shigemitsu H., Hamachi I. (2017). Design Strategies of Stimuli-Responsive Supramolecular Hydrogels Relying on Structural Analyses and Cell-Mimicking Approaches. Acc. Chem. Res..

[176] Qin Z., Yu X., Wu H., Li J., Lv H., Yang X. (2019). Nonswellable and Tough Supramolecular Hydrogel Based on Strong Micelle Cross-Linkings. Biomacromolecules.

[177] Yu B., Zhan A., Liu Q., Ye H., Huang X., Shu Y., Yang Y., Liu H. (2020). A designed supramolecular cross-linking hydrogel for the direct, convenient, and efficient administration of hydrophobic drugs. Int. J. Pharm..

[178] Che Y., Gaitzsch J., Liubimtsev N., Zschoche S., Bauer T., Appelhans D., Voit B. (2020). Double cross-linked supramolecular hydrogels with tunable properties based on host-guest interactions. Soft Matter.

[179] Le X., Lu W., Zhang J., Chen T. (2019). Recent Progress in Biomimetic Anisotropic Hydrogel Actuators. Adv. Sci..

[180] Xie R., Liang Z., Ai Y., Zheng W., Xiong J., Xu P., Liu Y., Ding M., Gao J., Wang J. (2021). Composable microfluidic spinning platforms for facile production of biomimetic perfusable hydrogel microtubes. Nat. Protoc..

[181] Adorinni S., Rozhin P., Marchesan S. (2021). Smart Hydrogels Meet Carbon Nanomaterials for New Frontiers in Medicine. Biomedicines.

[182] Zhang J., Liu Y., Chandra Sekhar P.D., Singh M., Tong Y., Kucukdeger E., Yoon H.Y., Haring A.P., Roman M., Kong Z. (2023). Rapid, autonomous high-throughput characterization of hydrogel rheological properties via automated sensing and physics-guided machine learning. Appl. Mater. Today.

[183] Owh C., Ho D., Loh X.J., Xue K. (2023). Towards machine learning for hydrogel drug delivery systems. Trends Biotechnol..

[184] Li F., Han J., Cao T., Lam W., Fan B., Tang W., Chen S., Fok K.L., Li L. (2019). Design of self-assembly dipeptide hydrogels and machine learning via their chemical features. Proc. Natl. Acad. Sci. USA.

[185] Wang Y., Wallmersperger T., Ehrenhofer A. (2024). Prediction of hydrogel swelling states using machine learning methods. Eng. Rep..

[186] Neufeld L., Yeini E., Reisman N., Shtilerman Y., Ben-Shushan D., Pozzi S., Madi A., Tiram G., Eldar-Boock A., Ferber S. (2021). Microengineered perfusable 3D-bioprinted glioblastoma model for in vivo mimicry of tumor microenvironment. Sci. Adv..

[187] Moghimi N., Hosseini S.A., Dalan A.B., Mohammadrezaei D., Goldman A., Kohandel M. (2023). Controlled tumor heterogeneity in a co-culture system by 3D bio-printed tumor-on-chip model. Sci. Rep..

[188] Datta P., Dey M., Ataie Z., Unutmaz D., Ozbolat I.T. (2020). 3D bioprinting for reconstituting the cancer microenvironment. NPJ Precis. Oncol..

[189] Abasalizadeh F., Moghaddam S.V., Alizadeh E., Akbari E., Kashani E., Fazljou S.M.B., Torbati M., Akbarzadeh A. (2020). Alginate-based hydrogels as drug delivery vehicles in cancer treatment and their applications in wound dressing and 3D bioprinting. J. Biol. Eng..

[190] Luo Y., Wei X., Wan Y., Lin X., Wang Z., Huang P. (2019). 3D printing of hydrogel scaffolds for future application in photothermal therapy of breast cancer and tissue repair. Acta Biomater..

[191] Kim D., Jo S., Lee D., Kim S.M., Seok J.M., Yeo S.J., Lee J.H., Lee J.J., Lee K., Kim T.D. (2023). NK cells encapsulated in micro/macropore-forming hydrogels via 3D bioprinting for tumor immunotherapy. Biomater. Res..

[192] Li J., Ji C., Lu B., Rodin M., Paradies J., Yin M., Kuckling D. (2020). Dually Crosslinked Supramolecular Hydrogel for Cancer Biomarker Sensing. ACS Appl. Mater. Interfaces.

[193] Wang W., Han R., Chen M., Luo X. (2021). Antifouling Peptide Hydrogel Based Electrochemical Biosensors for Highly Sensitive Detection of Cancer Biomarker HER2 in Human Serum. Anal. Chem..

[194] Mateti T., Likhith K., Laha A., Thakur G. (2023). A critical analysis of the recent developments in multi-stimuli responsive smart hydrogels for cancer treatment. Curr. Opin. Biomed. Eng..

[195] Solanki R., Bhatia D. (2024). Stimulus-Responsive Hydrogels for Targeted Cancer Therapy. Gels.

[196] Andrade F., Roca-Melendres M.M., Duran-Lara E.F., Rafael D., Schwartz S. (2021). Stimuli-Responsive Hydrogels for Cancer Treatment: The Role of pH, Light, Ionic Strength and Magnetic Field. Cancers.

[197] Sepantafar M., Maheronnaghsh R., Mohammadi H., Radmanesh F., Hasani-Sadrabadi M.M., Ebrahimi M., Baharvand H. (2017). Engineered Hydrogels in Cancer Therapy and Diagnosis. Trends Biotechnol..

